# Advances in pathogenesis and precision medicine for nasopharyngeal carcinoma

**DOI:** 10.1002/mco2.32

**Published:** 2021-01-07

**Authors:** Qian‐Ying Zhu, Ge‐Xin Zhao, Yan Li, Girish Talakatta, Hai‐Qiang Mai, Quynh‐Thu Le, Lawrence S. Young, Mu‐Sheng Zeng

**Affiliations:** ^1^ State Key Laboratory of Oncology in South China, Collaborative Innovation Center for Cancer Medicine, Guangdong Key Laboratory of Nasopharyngeal Carcinoma Diagnosis and Therapy Sun Yat‐sen University Cancer Center (SYSUCC) Guangzhou China; ^2^ Department of Radiation Oncology Stanford California; ^3^ Warwick Medical School University of Warwick Coventry United Kingdom

**Keywords:** Epstein‐Barr virus, nasopharyngeal carcinoma, precision medicine, signal transduction

## Abstract

Nasopharyngeal carcinoma (NPC) is a squamous carcinoma with apparent geographical and racial distribution, mostly prevalent in East and Southeast Asia, particularly concentrated in southern China. The epidemiological trend over the past decades has suggested a substantial reduction in the incidence rate and mortality rate due to NPC. These results may reflect changes in lifestyle and environment, and more importantly, a deeper comprehension of the pathogenic mechanism of NPC, leading to much progress in the preventing, screening, and treating for this cancer. Herein, we present the recent advances on the key signal pathways involved in pathogenesis of NPC, the mechanism of Epstein‐Barr virus (EBV) entry into the cell, and the progress of EBV vaccine and screening biomarkers. We will also discuss in depth the development of various therapeutic approaches including radiotherapy, chemotherapy, surgery, targeted therapy, and immunotherapy. These research advancements have led to a new era of precision medicine in NPC.

## Graphical abstract

Advances on the key signal pathways involved in pathogenesis of nasopharyngeal carcinoma (NPC), the mechanism of Epstein‐Barr virus (EBV) entry into the cell, the progress of EBV vaccine and screening biomarkers, and the development of therapeutic approaches were presented.

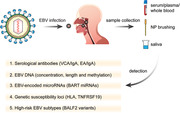

## 1 INTRODUCTION

Nasopharyngeal carcinoma (NPC), which arises from the epithelial lining of the nasopharynx, is frequently observed in the pharyngeal recess. Despite originating from a similar tissue lineage, there is a significant distinction between NPC and other mucosal head and neck cancers.^1^, ^2^

NPC is a complex disease involving genetic predisposition, infection with Epstein‐Barr virus (EBV), and environmental factors.^3^, ^4^, ^5^, ^6^, ^7^, ^8^ Compared to other cancers, NPC is not common. World widely, 129 079 cases of NPC were reported in 2018, accounting for only 0.7% of all types of cancers diagnosed in 2018. In particular, East and Southeast Asia are high prevalence areas, accounting for 70% of NPC cases.^9^, ^10^, ^11^ In China, the age­standardized incident rate by world (ASIRW) of NPC was 3.0 per 100 000, whereas it was 0.4 per 100 000 in Western countries.^12^, ^13^, ^14^ Notably, among seven administrative regions of China, the NPC incidence rate was significantly high in Southern China (9.69/100 000). In addition, the incidence of NPC among migrants from southern China is still high even when they live in nonendemic areas.^14^ Furthermore, demographic studies indicate that men are —two to three times more likely to develop NPC than women, in which the peak age of disease occurrence is greater than 45 years old.

Over the past two decades, the ASIRW of NPC has declined remarkably in East and Southeast Asia.^15^ This reduced incidence rate in NPC may be the results of promotion in healthy eating habits, economic growth, and restricted use of tobacco.^16^, ^17^, ^18^ Although there is no licensed EBV vaccine, it is deemed to be a promising strategy to prevent NPC. In addition to the decreased incidence, lower mortality rates are observed and are likely due to advances in screening and treatment for NPC.^19^, ^20^ Therefore, this review will focus on how precision medicine is impacting the prevention, screening, and treatment of NPC, and will also discuss the future prospects for the improved clinical management of NPC.

## 2 KEY SIGNAL PATHWAYS INVOLVED IN PATHOGENESIS OF NPC

**FIGURE 1:**
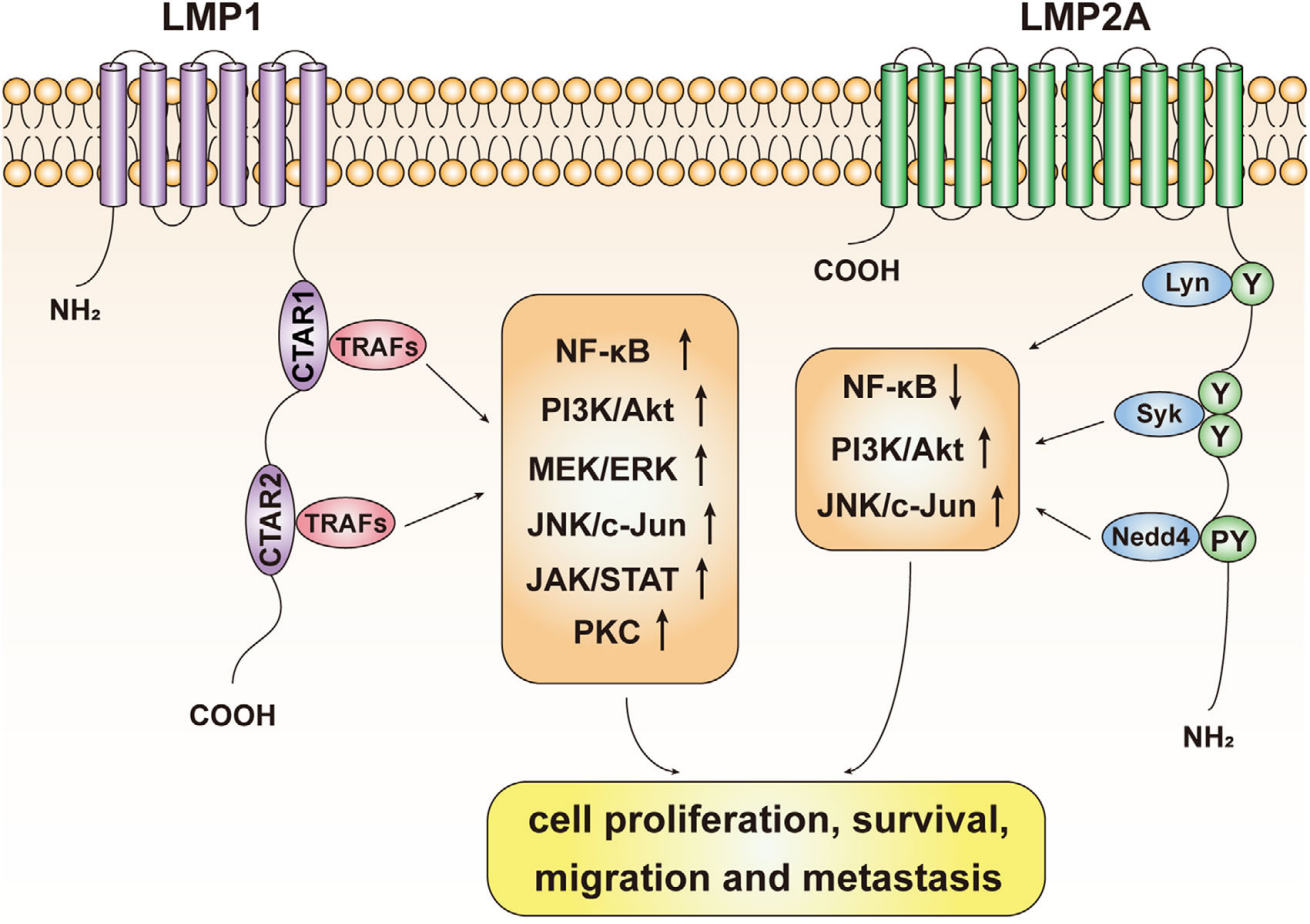
Signal pathways in nasopharyngeal carcinoma *Note*. LMP1 and LMP2A are two latent membrane proteins encoded by EBV. They play an important role in regulating several signal pathways including NF‐κB, PI3K/Akt, MEK/ERK, JNK/c‐Jun, JAK/STAT, and PKC. Tumor necrosis factor receptor‐associated factors (TRAFs) bind to the COOH‐terminal activation region (CTAR) of LMP1 and activate the key proliferation‐related signaling pathways. LMP2A could regulate signaling by its tyrosine kinase binding sites for Lyn, Syk, and Nedd4. Ultimately, these signals collectively promote cell proliferation, survival, migration, and metastasis.

EBV in NPC cells exhibits a type II latency infection, expressing a cluster of latent viral proteins and noncoding RNAs, which include latent membrane proteins (LMP1 and LMP2A/B), BamH1‐A fragment right ward reading frame 1 (BARF1), nuclear antigen (EBNA1), long noncoding RNAs (BARTs), small RNAs (EBER1 and EBER2), and microRNAs (miR‐BARTs).^21^ These viral latent genes, especially LMP1 and LMP2, play important roles in regulating several signaling pathways to promote tumor survival and metastasis, ultimately leading to a poor prognosis of NPC.^22^, ^23^, ^24^ The key pathways involved in pathogenesis of NPC are summarized in the following sections (Figure 1).

### 2.1 NF‐κB pathway

NF‐κB has two major functions in cells, namely, modulation of cell proliferation and regulation of the inflammatory response.^25^ NF‐κB signaling is constitutively activated in NPC tissues and is associated with poor prognosis.^26^, ^27^ In NPC, LMP1 can activate NF‐κB by binding to tumor necrosis factor (TNF) receptor‐associated factors (TRAFs), leading to the upregulation of several proliferative signals.^28^, ^29^, ^30^, ^31^ It has been demonstrated that LMP1 induces the interaction between telomerase reverse transcriptase (hTERT) and p65 (an NF‐κB subunit), resulting in telomerase activation and cell immortalization.^32^ Moreover, activation of this signaling pathway is also required for IL‐6, c‐Myc, and Bmi‐1 expression and the sustained cells proliferation of hTERT‐immortalized NPE cells.^33^

By contrast with LMP1, LMP2A downregulates the levels of NF‐Κb.^34^ As the important role of NF‐κB in inflammation regulation by inducing cytokine and chemokine expression, decline of this signaling pathway could inhibit the immune response against NPC.^35^ Thus, these contradictory effects of LMP2A and LMP1 in NF‐κB activation strike the balance between inflammation and apoptosis that ultimately promotes tumor proliferation. However, the interactions of these regulatory effects between LMP2A and LMP1 on NF‐κB signal pathway are yet to be fully clarified.

Recently, loss of function mutations on various negative regulators of the NF‐κB pathway in nearly half of NPC including NFKBIA, CYLD, TRAF3, TNFAIP3, and NLRC5 have been found by different groups using whole exome sequencing.^36^, ^37^, ^38^ These findings further strengthen the key role of the NF‐κB in NPC. It has been confirmed by functional analysis that both NFKBIA and CYLD inhibited the growth of NPC cells and that mutations in these genes resulted in NF‐kB activation. Interestingly, there are exclusive associations between high LMP1 expression and genetic defects of NF‐κB negative regulators, indicating that NF‐κB pathway could be activated by somatic mutations without LMP1 expression, further strengthening the crucial role of the NF‐kB pathway in the pathogenesis of NPC.

### 2.2 PI3K/Akt pathway

Studies have shown that the PI3K/Akt pathway is of great importance in EBV‐associated NPC.^39^, ^40^ The EBV‐activated PI3K/Akt pathway could promote pathogenicity via several mechanisms, including cell proliferation, apoptosis, genomic instability, and cytoskeleton dynamics.^41^, ^42^, ^43^ It has been reported that LMP1 promotes cell survival and migration through the activation of PI3K/Akt signaling in EBV‐positive NPC cell lines, and that these effects can be abolished after using the inhibitors of this pathway.^44^, ^45^, ^46^ These effects of LMP1 on the activation of PI3K/Akt are mediated by the COOH‐terminal activation region 1 (CTAR1) of region of the protein.^47^ Detections of activated PI3K in NPC offer further proof of the significance of this pathway for EBV carcinogenesis. By microarray and affymetrix assays, it has been found that in NPC biopsies, PI3K mediated the gene expression of Uridine diphosphate (UDP)‐glucose dehydrogenase induced by LMP2A, implicating the PI3K/Akt pathway in the increase in glucose input, which is required for cell proliferation.^48^, ^49^ In addition, Kong et al^50^ demonstrated that LMP2A induced epithelial to mesynchemal transition (EMT) and cancer stem cell phenotype by activation of PI3K/Akt pathway. Lee et al^51^ found that LMP1 could not only activate PI3K/Akt signaling pathway but also increase the expression of Bcl‐2, resulting in suppression of prostate apoptosis response‐4 (Par‐4) activity and its proapoptotic effect. In NPC cell lines, LMP1 inhibits DNA repair by activating PI3K/Akt pathway mediated by CTAR1. Sustained activation of FOXO3a has been reported to eliminate inhibition of DNA repair mediated by LMP1, whereas activation of PI3K/Akt would suppress the activity of FOXO3a.^52^ The cytoskeleton is so important in carcinogenesis that many target therapy is focusing on proteins involved in cytoskeleton regulation.^53^ In addition, it was reported that PI3K/Akt pathway is important in the formation of actin stress fibers and microtubule activity induced by LMP1.^47^ Lin et al found that activation of cdc2 mediated by LMP1 could phosphorylate the microtubules regulator Op18/stathmin.^54^ PI3K/Akt pathway may play a part in this process because it has been demonstrated to improve the activity of cdc2.^55^

### 2.3 RAS/RAF/MEK/ERK pathway

It has been demonstrated that the Ras/Raf/mitogen‐activated protein kinase (MEK)/extracellular signal‐regulated kinase (ERK) signaling pathway regulates many different biological processes and plays an important role in intercellular and intracellular communication.^56^, ^57^, ^58^ Upregulation of this pathway is associated with a variety of tumors, including gastric adenocarcinoma, hepatocellular carcinoma, and NPC.^59^, ^60^, ^61^ In epithelial cells, LMP1 behaves as a TNF receptor, constitutively activating RAS and the canonical RAF/MEK/ERK pathway, which may participate in some transforming effects such as EMT in NP epithelial cells (NPECs).^62^ Dawson et al showed that induction of the EMT phenotype by LMP1 required functional CTAR1 domain, the TRAF binding domain that was previously implicated in PI3K activation.^63^ Additionally, LMP1 can activate RAS via inducing epigenetic alterations in its negative effectors. Moreover, the downregulation of RASSF proteins was found in 80% of NPC cell lines. Pharmacological inhibition of this pathway could lead to the reversal of the EMT phenotype in cells with high LMP1 expression.^64^, ^65^ Multiple studies show that high ERK levels in NPC are associated with poor prognosis.^21^, ^66^, ^67^ Therefore, among several mechanisms involved in EMT, the activation of this pathway contributes to LMP1‐mediated EMT thereby enhancing cell motility and invasive properties.^66^

### 2.4 JNK/c‐Jun pathway

In addition to ERK, the c‐Jun N‐terminal kinases (JNKs) have been well studied in NPC.^68^, ^69^, ^70^ Abnormal activation of JNK pathways has been reported in various cancers including NPC, showing that JNK plays a key role in tumorigenesis and controls some basic features of cancer cells such as proliferation, apoptosis, and migration.^71^, ^72^, ^73^ In EBV‐positive NPC, the activating effect of LMP1 on the JNK pathway has been clearly demonstrated. And studies suggested that JNK activation can increase p53 phosphorylation and activation of DNA methyltransferase (DNMT), which leads to p53 inactivation, DNA hypermethylation, and inhibition of E‐cadherin expression.^73^, ^74^, ^75^ LMP2A is also found to have the ability to activate the JNK pathway and induce hyperphosphorylation of the downstream effector c‐Jun.^76^ Indeed, Zhang and his team reported that over activation of JNK is related with development and progression of NPC, where higher expression of c‐Jun is positively correlated with more advanced tumor stage.^68^, ^74^

### 2.5 JAK/STAT pathway

As a cytoplasmic transcription factor, the signal transducer and activator of transcription (STAT) protein could relay signal from receptors of growth factor and cytokine on the cell surface to the nucleus, regulating cell proliferation, apoptosis, and differentiation.^77^, ^78^, ^79^, ^80^ STAT3 and STAT5 are most relevant to NPC among the STAT family members.^81^, ^82^, ^83^ LMP1 can increase the transcription, phosphorylation, and nuclear translocation of STAT by upregulating the expression of IL‐6.^84^, ^85^, ^86^ In turn, the STAT pathway could regulate LMP1 expression in NPC cells. There is an automatically regulated positive feedback loop, in which STAT‐induced LMP1 expression leads to upregulation of IL‐6, which itself results in STAT activation and subsequent induction of LMP1 and EBNA1 expression.^85^ JAK/STAT3 pathsway activation leads to antiapoptotic effects with concomitant proproliferative and migrative effects.^87^ In addition, STAT3 is also implicated in angiogenesis and regulation of EBV latent infection, contributing to the highly aggressive characteristic of NPC.^77^, ^85^, ^88^, ^89^ STAT3, a direct transcriptional activator of the vascular endothelial growth factor (VEGF) gene, can induce VEGF expression and ultimately enhances the capacity of migration and invasion of NPC cells.^90^ In addition, LMP1‐mediated activation of JAK/STAT pathway is rapid, suggesting that it may proceed NF‐κB and JNK activation and predispose the cell to these later signals.^84^, ^86^, ^91^

### 2.6 PLC/PKC pathway

Protein kinase C (PKC) includes a family of phospholipid‐dependent serine/threonine kinases, whose activation results in the phosphorylation of certain proteins that can contribute to cell proliferation, apoptosis, and differentiation.^92^, ^93^ The PKC can be activated by ligands of TNFR family, such as TNFα and CD40. In NPC cells, LMP1 functions as a TNFR member‐like molecule and it can activate multiple signaling pathways including the PKC signaling pathway.^94^ Annexin A2 is a 36‐kDa protein that acts as an intercellular transport protein, interfering in multiple cellular processes including cell division and migration noted in cancer progression.^95^ LMP1 not only upregulates the level of phosphorylation of annexin A2 but also induces its nuclear translocation via the PKC pathway.^94^ Upon phosphorylation, annexin A2 enters the nucleus and promotes DNA synthesis and cell proliferation.^96^, ^97^ In addition to annexin A2, LMP1 is also involved in the phosphorylation of Ezrin through the PKC pathway. Ezrin, a membrane crosslinker protein, has been implicated in the migration and invasion of NPC cells.^98^ Furthermore, the PKC pathway is considered essential for activating cells latently infected with EBV into the virus lytic cycle. As a PKC agonist, the phorbol ester tetradecanoylphorbol acetate (TPA) is a well‐known activator of EBV lytic replication.^99^

## 3 PROGRESS IN PREVENTION OF NPC

The pathogenesis of NPC is intimately associated with EBV infection. As we know, EBV could be detected in nearly 99% of nonkeratinizing NPC, which is the most common histological subtype in endemic regions.^19^, ^100^ Therefore, an EBV prophylactic vaccine has been touted as a potential means to prevent this disease.^101^, ^102^, ^103^ However, despite decades of research, there is no licensed EBV vaccine available for use so far. Understanding how EBV enters the cells is crucial to developing an effective vaccine.^104^ In this section, we will summarize the mechanism of EBV infection and the progress of EBV vaccine development.

### 3.1 EBV infection of epithelial cells

**FIGURE 2:**
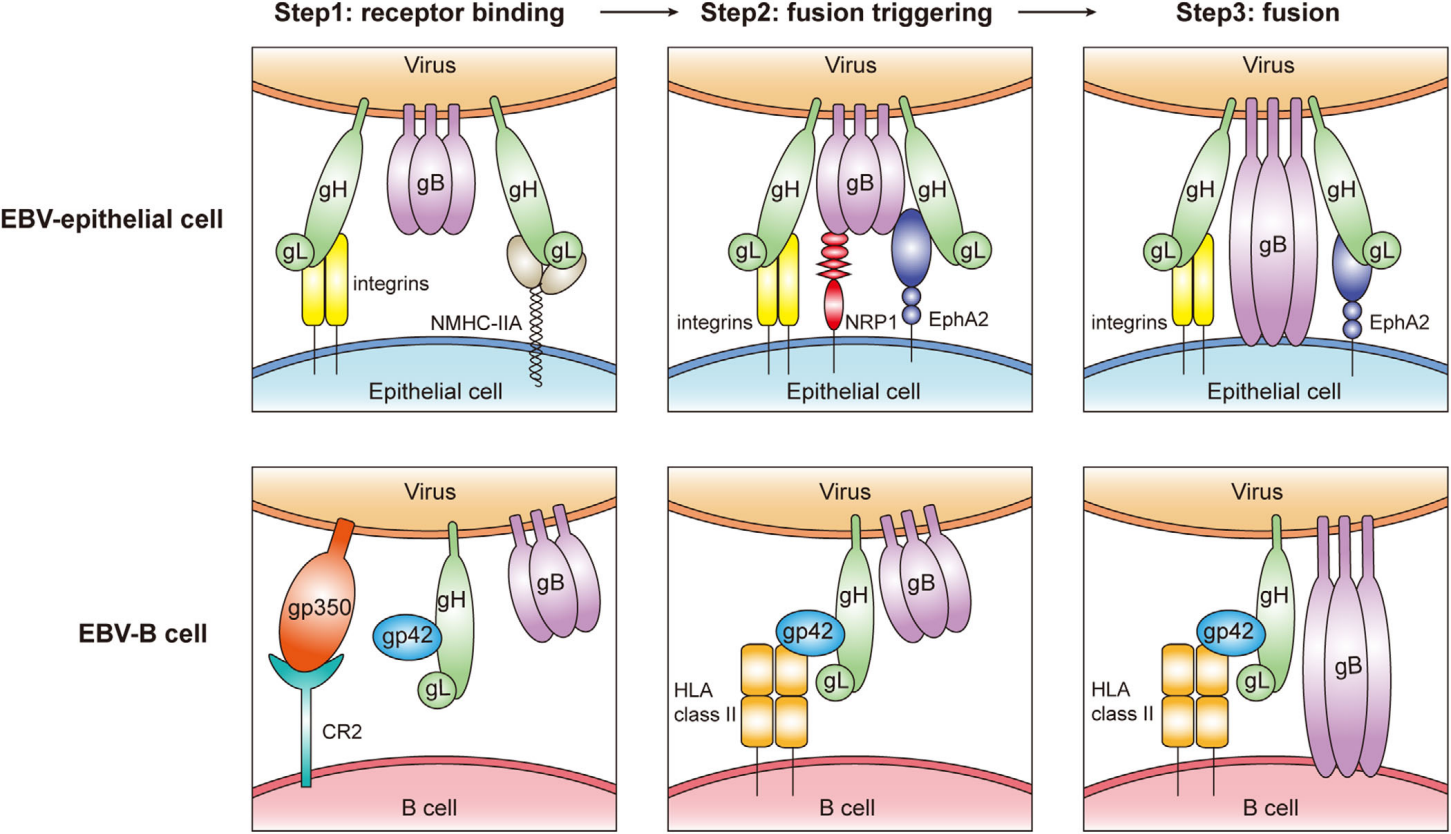
Mechanisms of EBV infection of epithelial cell and B cell *Note*. In the progress of EBV infection into epithelial cells, EBV glycoprotein gH/gL binds to integrin and NMHC‐IIA on the cell surface first. Then gH/gL/gB complex interacts with NRP1 and EphA2, triggering the conformational transition of gB. Lastly, the fusion loops of gB insert into the cell membrane and complete the fusion of EBV. At the first step of EBV infection into B cells, gp350 binds to its receptor protein CR2. After that, interaction between gp42 and HLA class II triggers the fusion progress. Eventually, gH/gL and postfusion gB drive the membrane fusion of EBV and B cell.

Glycoproteins on the cell surface of the EBV are crucial for its entry into host cells^105^, ^106^, ^107^ (Figure 2). Compared to epithelial cells, the interaction of EBV with B cells is more thoroughly understood. EBV first utilizes gp350, one of the most abundant glycoproteins in the viral envelope, to bind to CD21 or CD35 expressed on the B‐cell surfaces.^108^, ^109^ Then the binding of gp42 to HLA class II triggers the EBV‐cell fusion. Finally, with the help of gH/gL (gL is the chaperone for gH) and gB, EBV completes the fusion with B cells. It is widely accepted that gH/gL and gB are key fusion components as evidenced by the impaired fusion when either gH/gL or gB is deleted or mutated.^110^, ^111^, ^112^, ^113^

Because CD21 expression is low on the surface of epithelial cells, it is speculated that gp350 is not important for EBV binding to these cells.^114^, ^115^ Furthermore, treatment with 72A1 antibody that binds gp350, which efficiently blocks EBV infection of B cells, actually increases epithelial infection.^116^, ^117^ In contrast, gp350‐null EBV infects both B cells and epithelial cells less efficiently than wild‐type EBV, suggesting that gp350 may somehow play a part in EBV epithelial infection.^118^ One possible explanation of this observation is that gp350 may bind to cell surface molecules other than CD21 in epithelial cells. In fact, there is data to suggest that other cell surface proteins that interact with EBV glycoproteins on B cells may not be expressed on epithelial cells. For example, the HLA class II, which functions as gp42 binding receptor in B cells, is completely absent in epithelial cells.^119^, ^120^ Gp42‐null EBV, which fails to infect B cells, successfully infects epithelial cells, indicating that gp42 is not required for epithelial infection. Interestingly, gp42 was found to suppress EBV infection of epithelial cells.^121^

Previous studies have showed that EBV lacking gH failed to infect both B cells and epithelial cells.^122^ However, antibody F‐2‐1, which targets gH, inhibits the ability of EBV to infect B cells but not epithelial cells.^122^ In contrast, another antibody E1D1, which also targets gH, has no effect on EBV infection of B cells but impairs its infection of epithelial cells.^123^ It is noteworthy that a monoclonal anti‐gH/gL antibody, AMMO1, could simultaneously inhibit EBV infection of both epithelial and B cells.^124^ These results demonstrate the important but different roles that gH plays in EBV infection of B and epithelial cells. The entrance of enveloped virus into host cells can be divided into two steps: (a) viral binding to the cell surface and (b) viral penetration and fusion with the cell membrane to release its capsid and nucleus into the cytoplasm.^125^ EBV gH primarily mediates EBV penetration into B cells, but it appears to be involved in both the viral binding and penetration into epithelial cells. A serial studies from the Hutt‐Fletcher group found that gH/gL binding to integrin αvβ5, αvβ6, and αvβ8, but not αvβ3 could trigger fusion.^126^, ^127^ Either knockdown of αv by siRNA or exposure to the soluble αvβ6 and αvβ8 to culture media lead to decreased EBV infection efficiency of epithelial cells. Adding soluble αvβ5, αvβ6, or αvβ8 triggered the fusion of Chinese hamster ovary cell (CHO)‐K1 cells transfected with gB and gH/gL. However, another group found that knockout of αv in HEK293 cells had no effect on EBV fusion or infection.^128^ One possible reason for the inconsistent results might be the using of different cell lines. However, it is likely that there are other host cell surface proteins that can interact with gH/gL in addition to integrins.

The extremely low EBV infection efficiency in primary nasopharynx epithelial cells raises questions of how and when EBV enters its host cells in NPC patients. In normal NP epithelia, EBV was rarely detected, but it was readily seen in stratified dysplastic nasopharynx epithelia, which is a precancerous state of NPC.^129^ Xiong and colleagues^116^ have recently established an efficient EBV infection model using BMI1‐immortalized NPECs to form multilayered sphere‐like cells (SLCs), which are highly susceptible to EBV infection. BMI1‐immortalized NPECs also have precancerous features, such as low expression of p16 and high activity of telomerase.^130^, ^131^ These results indicate that SLCs may resemble the NPC precancerous state. Nonmuscle myosin heavy chain IIA (NMHC‐IIA) was pulled down by myc‐tagged gH/gL proteins in the SLC lysates, and identified by liquid chromatography‐mass spectrometry (MS)/MS. EBV infection of SLCs was inhibited by the NMHC‐IIA downregulation or NMHC‐IIA antibody blocking, suggesting that NMHC‐IIA is a key and fundamental host factor mediating EBV infection of SLCs. Interestingly, NMHC‐IIA protein is located in the cytoplasm of NPEC monolayer cells (MLCs), which was fixed to SLCs membrane to interact with gH/gL. Much high level of NMHC‐IIA was detected in NP dysplastic epithelium than normal counterpart, indicating that cells with high levels of this protein are more susceptible to EBV infection.

While deletion of gB completely inhibited virus production, high levels of gB resulted in higher infection efficiencies.^132^, ^133^ Unfortunately, gB‐null EBV has not been generated despite significant effort. The highly conserved gB is required for membrane fusion of herpes viruses.^134^, ^135^, ^136^, ^137^, ^138^, ^139^ Wang et al^140^ recently found that neuropilin 1 (NRP1) could interact with gB, acting as an EBV entry factor in NPECs. Interestingly, downregulation of NRP1 inhibited EBV infection of NPECs, but downregulation of NRP2 promoted the infection. Using binding and entry assays, it was found that NRP1 had no effect on EBV binding to NPECs, but affected EBV fusion and internalization into NPECs.

It is well known that growth factors and cytokines play important roles in cancer initiation, development, and progression. Viral infection may be influenced by cytokines or itself may induce cancer promoting cytokines. Zhang et al found that EGF and several cytokines could enhance EBV infection of NPECs.^141^ In EGF‐treated NPECs, six membrane protein‐encoded genes were highly induced, including EPHA2, EGFL5, F3, AREG, DCBLD2, and NT5E. Among these genes, EBV infection was significantly inhibited by EphA2 downregulation. Similarly, EBV infection was inhibited by treatment with anti‐EphA2 antibody. EphrinA1 and 2,5‐dimethylpyrrolyl benzoic acid derivatives, which were the ligand and antagonist of EphA2, could also inhibit EBV infection. As mentioned above, gH/gL and gB function as core fusion machinery. Surprisingly, EphA2 interacts with gH/gL and gB simultaneously, which is different from NMHC‐IIA and NRP1. The ectodomain of EphA2 includes three parts: the Ephrin‐binding domain (EBD), the cysteine‐rich domain (CRD), and the Fibronectin III type repeats (FNRs).^142^ Both gH/gL and gB interacted with the EBD domain, but gB also interacts with the FNR domains. Further, it was demonstrated that EphA2 promoted the internalization and fusion but not binding of EBV. Since B cells lack EphA2 expression, the promotion of EBV infection by EphA2 is specific to epithelial cells. Consistently, by comparing B cell and HEK293 cell RNA‐seq dataset, Chen et al also identified EphA2 as the epithelial receptor for EBV infection.^128^

Although EBV is rarely detected in normal NP epithelium, the primary infection that occurs in polarized columnar epithelium cannot be ruled out. By studying EBV infection of polarized tongue and NPECs, EBV envelope protein BMRF2 was found to bind integrin αvβ1.^143^ Most of the epithelial cell lines are refractory to EBV infection when virus is simply added to the culture medium, so‐called cell‐free (cf) infection.^144^ However, cocultured with EBV‐positive B cells, it significantly improves the efficiency of infection, a process known as cell‐to‐cell infection.^144^, ^145^, ^146^ Treatment with blocking antibodies of the β1 and β2 integrin families and their ligands inhibit EBV cell‐to‐cell transmission. Interestingly, in the process of EBV infection of epithelial cells, in‐cell infection was also observed.^147^, ^148^ Ni et al^146^ reported that cell‐in‐cell structures mediate the process of EBV transmission from B cells to epithelial cells. And EBV produced by infected epithelial cells showed higher infection efficacy to both epithelial and B cells.

The extremely low EBV infection efficiency in primary nasopharynx epithelial cells raises questions of how and when EBV enters its host cells in NPC patients. Studies on the mouse gammaherpesvirus 68 suggest that neonatal infection of the mouse results in respiratory epithelial infection, whereas infection in the adult mouse results in an “infectious mononucleosis” (IM)‐like syndrome.^149^ Similarly, in humans, it would appear that in the susceptible populations for NPC (China, Southeast Asia), EBV infections appear to occur early in life, whereas in populations with low incidence of NPC (Caucasians), EBV infections appear later in the teens and which incidentally appears to also be associated with a higher incidence and a bimodal peak of EBV‐related Hodgkin's lymphoma. Interestingly, in cities like Hong Kong and Singapore, where the incidence of NPC has been in the decline in the last few decades, the incidence of Hodgkin's lymphoma in those 2 cities is slowly picking up and beginning to mimic the bimodal peak that was found in the Caucasian countries.^150^ Other infection‐related cancers like Hepatitis B‐associated hepatocellular carcinoma, H‐Pylori‐related gastric carcinoma, and even Merkel Cell Carcinomas have in common either perinatal, neonatal, or early infections. Few if any of the studies looking at viral infections utilize neonatal models, and if the physiology of the neonate is significantly different from the adult, then one might be forgiven for wondering if the conclusions thus drawn may perhaps be significantly compromised.

### 3.2 EBV prophylactic vaccines trials in humans

Scientists strongly endorsed the important goal of conducting vaccine trials to prevent not only IM but also a series of EBV‐related cancers.^151^ On the cell surface of EBV virions, there are many glycoproteins, including gp350, gH/gL, gB, gp42, and so on, which could serve as vaccine targets. Among these glycoproteins, the most abundant one is gp350. Therefore, gp350 was considered the most promising immunogen for EBV vaccine so that it has been most extensively studied as a vaccine immunogen leading to some EBV vaccine trials^152^, ^153^ (Table 1).^101^

**TABLE 1:** Clinical trials for prophylactic EBV vaccines

Vaccine	Adjuvant	Study subjects	Phase	Size	Result	Reference
Vaccinia‐gp350	None	EBV and vaccinia virus‐naive infants aged 1‐3 years	I	9	Induced neutralizing antibody; may have reduced infection.	Gu et al^154^
Recombinant gp350	None, alum, or alum/MPL	EBV‐seronegative adults aged 18‐37 years	I/II	81	Induced neutralizing antibody	Moutschen et al^155^
Recombinant gp350	Alum/MPL	EBV‐seronegative volunteers aged 16‐25 years	II	181	Induced neutralizing antibody; was efficient in preventing IM but not EBV infection	Sokal et al^156^
Recombinant gp350	Alum	EBV‐seronegative children with CKD awaiting renal donation	I	13	Induced transient neutralizing antibody in some recipients, but could not prevent PTLD	Rees et al^157^
EBNA3 peptide	Tetanus toxoid in oil and water emulsion	EBV‐seronegative, HLA B*0801‐positive volunteers aged 18‐50 years	I	14	Induced EBV‐specific CD8+ T‐cell response, may prevent IM	Elliott et al^158^

Abbreviations: Alum, aluminum; MPL, monophosphoryl lipid A; EBNA3, Epstein‐Barr virus nuclear antigen 3; CKD, chronic kidney disease; HLA, human leukocyte antigen; IM, infectious mononucleosis; PTLD, posttransplant lymphoproliferative disorders.

Gu et al^154^ conducted the first EBV vaccine trial in China. Subjects were vaccinated with gp350 expressing vaccinia virus. No elevated level of EBV‐neutralizing antibody was observed in adults who were vaccinia virus‐seropositive and EBV‐seropositive. However, in children who were vaccinia virus‐seronegative but EBV‐seropositive, increased level of EBV antibody was detected. Vaccination of nine infants (aged 1‐3) who were both vaccinia virus‐seronegative and EBV‐seronegative led to a high level of EBV‐neutralizing antibody. During a follow‐up of 16 months, all unvaccinated controls infected with EBV, while only one‐third of vaccinated children became infected. Though encouraging, the numbers of participants in this trial were still too small to prove its efficacy and the toxicity of live vaccinia virus precluded large‐scale trials.

According to a phase I/II study, vaccines of recombinant gp350 produced by CHO cells could induce EBV‐neutralizing antibodies.^155^ Compared with subjects receiving soluble gp350 alone, recipients vaccinated with gp350 in adjuvant of alum/monophosphoryl lipid A (MPL) had higher level of neutralizing antibody against EBV. There was one serious adverse event that was thought to be related to the vaccine. Symptoms of headache, meningismus, and polyarthritis occurred in one recipient vaccinated with recombinant gp350 in alum/MPL but were resolved within 2 months.

Another phase II trial with 181 EBV‐seronegative volunteers was conducted to evaluate the performance of gp350 vaccine.^156^ Recipients were vaccinated with either placebo (adjuvant alone) or recombinant gp350 in alum/MPL in a three‐dose regimen (0, 1, and 5 months). Nearly all subjects with gp350 vaccination produced detectable antibody to gp350 one month after the last dose and maintained positive antibody titers for more than 18 months. Although the vaccine reduced the rate of IM by 78% in the vaccinated subjects, it did not produce the sterilizing immunity necessary to prevent persistent EBV infection.

A phase I trial was performed to evaluate the effect of the gp350 vaccine on preventing posttransplant lymphoproliferative disorders (PTLDs).^157^ Sixteen EBV‐seronegative children were recruited according to the eligibility criteria for kidney transplantation due to chronic kidney disease. Subjects were given three doses of gp350 in alum and all 13 recipients developed an anti‐gp350 Immunoglobulin G (IgG) response, but neutralizing antibodies could only be detected in four recipients. Of note, immune responses fell rapidly, and one patient developed PTLD with transplantation 50 weeks after the first dose of vaccine. Hence, the authors concluded that coordination is needed to complete all doses of vaccine before transplantation and a more suitable adjuvant is needed for the next trial.

A phase I study of randomized, single‐blind, placebo‐controlled, two‐dose EBV peptide vaccine was performed in EBV‐seronegative adults with HLA B*801.^158^ Subjects received EBNA‐3A peptide with tetanus toxoid in a water‐in‐oil adjuvant or placebo. None of the 10 vaccine recipients developed IM, while one of four placebo subjects developed IM. Asymptomatic EBV infection occurred in 4 of the 10 vaccine recipients and in one of the four placebo subjects. In eight of the nine vaccinated subjects, EBV‐specific T cell response was detected, none of them showed any adverse events.

### 3.3 Novel strategy for EBV vaccines

The EBV prophylactic vaccines showed that gp350 vaccines can reduce the occurrence of IM, but cannot effectively prevent EBV infection.^102^ These results may be due to the low titer of neutralizing antibodies induced by the gp350 vaccine alone, which cannot completely block the viral infection and by the need to generate a local mucosal immune response to block the initial infection. Therefore, vaccine improvement strategies through either improving the adjuvants or incorporating protein polymers such as virus‐like particle (VLP) or nanoparticles were evaluated.^159^, ^160^, ^161^, ^162^, ^163^, ^164^ Moreover, developing a vaccine to combination of gB, gH/gL, gp42, and other proteins may increase the diversity of neutralizing antibodies to prevent EBV infection.^165^

In 2015, Ogembo et al constructed an EBV gp350/220 chimeric VLP vaccine. They found that persistent neutralizing antibodies were produced in VLP‐immunized mice.^166^ But the serum antibody titer of EBV gp350/220‐VLP was slightly lower than that of the UV‐EBV control group. The immunogenicity of VLP particles may be reduced by the insertion of an exogenous gene that caused misfolding of the EBV surface proteins.

To enhance the antigenic immunogenicity of gp350, Kanekiyo et al used ferritin and encapsulin, two self‐assembling particles, to display D123 of gp350.^167^ Immunization of mice and nonprimates with constructed gp350‐D123‐ferritin and gp350‐D123‐encapsulin nanoparticles led to a 10‐ to 100‐fold higher level of neutralizing antibodies than vaccination with the soluble form of gp350 alone. The authors speculated that nanoparticle may help gp350 antigen to activate B cells, thus improving vaccine‐induced immunity.

Recently, Zhao et al found that constructing a dimer antigen form of Fc‐based gp350 could also improve the effectiveness of vaccination.^168^ They found that the gp350‐Fc fusion protein was more immunogenic than the wild‐type gp350 protein. The fusion of gp350‐ectodomainor residues 1‐425 (D123) with the mouse IgG2a Fc domain did not affect the folding of gp350. Compared with gp350 monomer, gp350‐Fc dimer significantly increased anti‐gp350 antibody titers and neutralizing antibody against EBV.

The above findings suggest that other EBV virion antigens may be needed for successful prophylactic vaccination. Tetrameric/monomeric gp350‐D123, trimeric/monomeric gH/gL, and trimeric gB proteins were produced in CHO cells and used for immunization in rabbits.^169^ Comparing the effect of different vaccination strategies, surprisingly, all of the other immunogens could induce higher EBV‐neutralizing titers than monomeric gp350‐D123.

Perez et al^170^ successfully produced gH/gL‐EBNA1 and gB‐LMP2 VLPs in CHO cells and immunized BALB/c mice without adjuvant. In spite of generating high neutralizing antibody level, such VLPs could also elicit EBV‐specific T‐cell responses in vivo. The authors suggested that triggering cell‐mediated immunity to EBV latent proteins, such as LMP2 and EBNA1, should be considered when designing EBV prophylactic vaccines.

Recently, Bu and his team^165^ designed an EBV vaccine with nanoparticles displaying gH/gL or gH/gL/gp42. They tested this vaccine in mice and nonhuman primates and found that both nanoparticles could elicit high level of neutralizing antibody to prevent EBV infection of epithelial cells and B cells. Moreover, compared to gH/gL‐ferritin, gH/gL/gp42‐ferritin induced stronger B‐cell neutralizing titers. From their results, gH/gL/gp42‐ferritin is a promising EBV vaccine candidate.

It is difficult to conduct a clinical trial to evaluate the effectiveness of a prophylactic EBV vaccine in reducing the incidence of NPC or other EBV‐associated cancers due to the long latency period from primary EBV infection to cancer development. However, lessons from the generation and use of vaccines against human papilloma virus (HPV) and human hepatitis B virus (HBV) indicate that the development of an effective EBV vaccine would be facilitated by the use of surrogate markers such as neutralizing antibodies titer. Cooperation between academic, industry, and government organizations is needed to accelerate EBV vaccine development.^171^, ^172^

## 4 PRECISION SCREENING AND DIAGNOSIS FOR NPC

**FIGURE 3:**
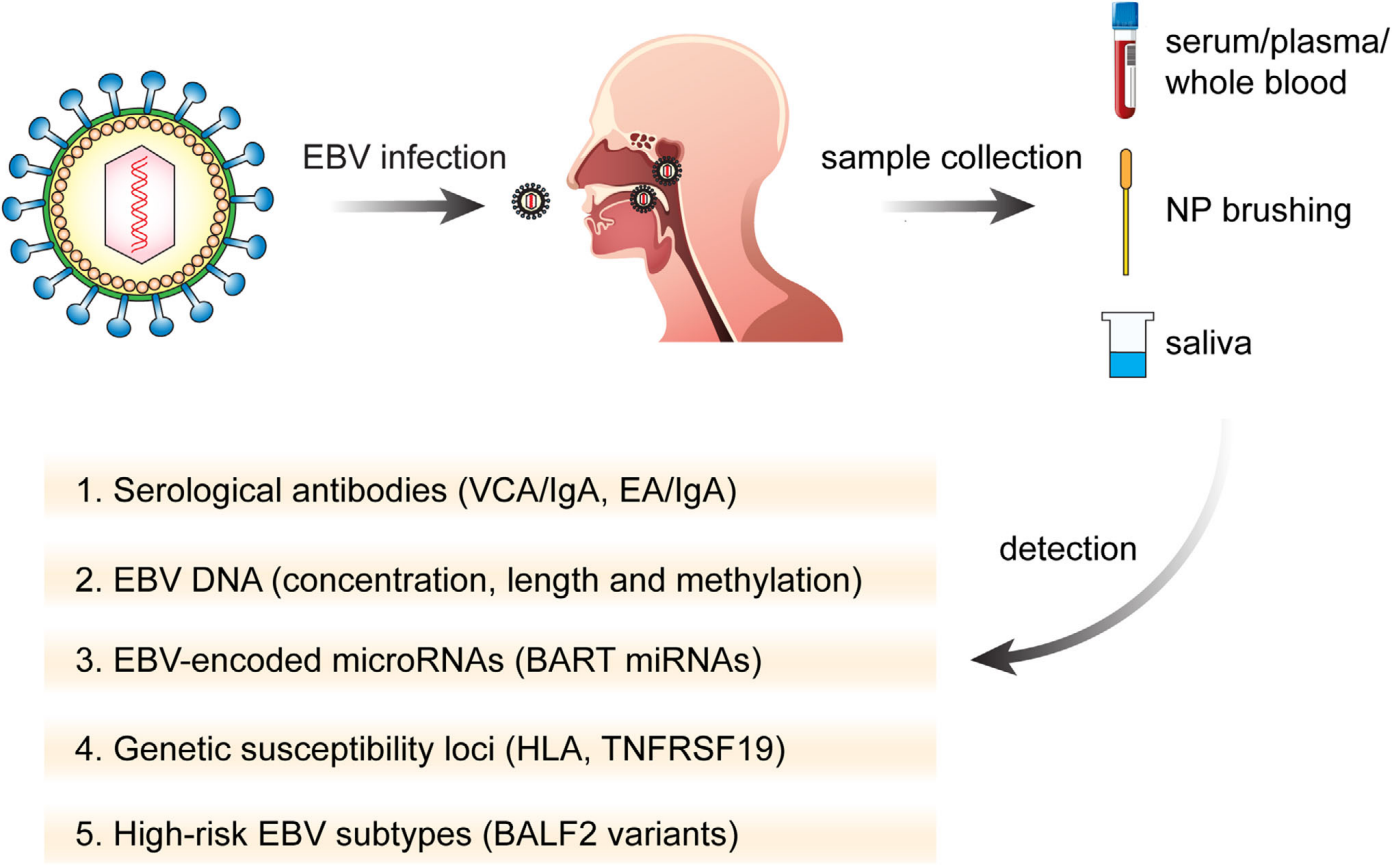
Strategies for the screening of nasopharyngeal carcinoma *Note*. EBV‐related markers are the most useful biomarkers in early screening for NPC. Samples for screening could be serum, plasma, whole blood, NP brushing, and saliva. Serological antibodies, such as VCA/IgA and EA/IgA, are the most widely used in the population‐based screening. In addition, EBV DNA is a good indicator with high sensitivity and specificity. Notably, EBV‐encoded microRNAs, genetic susceptibility loci, and high‐risk EBV subtypes are also helpful for comprehensive screening and should be taken into consideration.

Because of the hidden position of the nasopharynx, the early symptoms of NPC are not obvious and are therefore easily ignored by patients, leading to missed opportunities for early diagnosis. When symptoms and signs such as cervical lymph node enlargement, progressive nasal obstruction and bleeding, and stuffed ear occur, the patients frequently (up to 85%) present with more advanced NPC. Since the treatment of early stage of NPC is very successful with a significant 5‐year survival rate (from 70% to 90%), early detection through screening is crucial to decrease the mortality rate of NPC and this could be accomplished using EBV‐related biomarkers (Figure 3).^2^, ^3^, ^173^, ^174^, ^175^

### 4.1 Serological antibody against EBV

The titer of antibodies against certain EBV protein is higher in NPC patients compared to healthy people and varies during the development of the disease. Certain serologic markers have been widely used for screening and early diagnosis in high‐risk areas of NPC. In the 1970s, immunofluorescence detection of EBV capsid antigen (VCA) and early antigen (EA) in serum was used to screen for NPC. This technology represented the first generation of serological detection method and quickly became the gold standard for the screening of NPC. However, this traditional method depends on manual labor and has many drawbacks, such as low efficiency, low reliability, and a high rate of missed detection. These limitations were overcome by the subsequent development of enzyme‐linked immunoabsorbent assay (ELISA), which is a more accurate and automated second‐generation serological technique, and provides quantitative data. Thus, the ELISA technique is more suitable for large‐scale population screening.^176^, ^177^

To date, large‐scale prospective studies have been conducted in Guangdong and Taiwan, confirming that antibodies against certain EBV proteins are useful screening for NPC screening.^178^, ^179^, ^180^ Immunoglobulin A (IgA) antibodies to VCA (VCA/IgA) have high sensitivity, while IgA antibodies to EA (EA/IgA) have greater specificity. Thus, combination of these two markers is thought to be a suitable screening tool for NPC. Moreover, a sustained increase in the titers of both antibodies also suggests a high risk for NPC. Notably, the positive rate of VCA/IgA in NPC is only 70‐95%, thereby missing up to 5‐30% of NPC patients. Liu et al used VCA/IgA combined with EBNA1‐IgA to screen 5481 subjects in a South China endemic area, Sihui County, and found that the sensitivity of the combined test was only 75%.^181^ To improve detection sensitivity, Li et al combined gH/gL‐IgA with VCA/IgA and found that this combination yielded better results.^182^ However, the sample size in this study was relatively small, and larger scale cohort analysis in a multicenter study is required to confirm its diagnostic significance.

These selected antibodies for NPC diagnosis such as VCA, EA, and EBNA1 represent only a small part of the immune response to nearly 100 proteins encoded by EBV. Whether detection of antibodies against other EBV proteins can be used for NPC diagnosis is unclear. To answer this question, Coghill and his team used a custom protein microarray to detect the level of IgG and IgA antibodies to nearly 200 EBV proteins in three independent Taiwan cohorts.^183^ Comparing antibody levels in stage I/IIa NPC patients and control groups, they found that 73 IgG and 60 IgA anti‐EBV antibodies were different. After evaluation in cross‐sectional data, 12 promising antibody targets were identified. The authors combined these 12 biomarkers with VCA‐IgA and EBNA1‐IgA to develop a new and useful model for NPC prediction. This model could increase the prediction accuracy to 93%, which is higher than that of VCA/EBNA1 alone (82%) in the general Taiwanese population. For genetically high‐risk families, the prediction ability was improved to 89% with this risk score compared to 78% for current biomarkers.

### 4.2 EBV DNA

During the predominantly latent virus infection observed in NPC, EBV exists in cells in the form of extrachromosomal circular DNA that is shed into the blood circulation as tumor cells proliferate and undergo cell death. The majority of cf plasma EBV DNA fragments are between 82 and 181 kb in size, so quantitative polymerase chain reaction (QPCR) amplification can be used to detect the content of cf DNA in plasma.^184^, ^185^, ^186^ PCR studies have targeted different segments of the EBV genome including EBNA‐1, EBNA‐2, BXLF‐1 gene, and Bam HI‐W region. Among them, the Bam HI‐W region is the most commonly used region because of its great sequence conservation and repeatability.^187^, ^188^

In 1998, Mutirangura et al first found that QPCR could detect EBV DNA in the serum of 30% of NPC patients.^189^ In 1999, Lo and his colleagues confirmed that the level of EBV DNA in plasma was significantly related to stage, recurrence, and prognosis of NPC. They suggested that EBV DNA is a promising biomarker of diagnostic and prognostic evaluation of NPC.^190^ Since then, multiple studies have found that the level of cf EBV DNA is positively correlated with tumor load in vivo, which provides tremendous assistance in the management for NPC. In 2017, Chan et al performed a large‐scale and prospective cohort study in Hong Kong to assess the effectiveness of plasma EBV DNA in screening for NPC.^191^ A total of 20 174 otherwise healthy Chinese men who were 40 to 62 years of age had plasma EBV DNA measurement. Those with positive results had a second EBV DNA measurement about 4 weeks later. Magnetic resonance imaging and nasopharyngoscopy examination were performed for those whose EBV DNA was consistently positive twice. Thirty‐four cases of NPC were found, 71% of which were in the early stage, with a positive predictive value (PPV) of 11%. In 2018, using target‐capture sequencing technique, the same group found that the fragment lengths of plasma EBV DNA of NPC are longer than those of non‐NPC subjects.^192^ By measuring both the concentration and the length of EBV DNA, the PPV for NPC was improved to 19.6%, an improvement on previous results (11.0%) from their population screening study. In 2019, this group further performed whole‐genome methylation analysis of plasma EBV DNA fragments between NPC and non‐NPC subjects demonstrating differential methylation patterns. When combining the methylation pattern with concentration and fragment lengths of plasma EBV DNA, they found that a PPV of 35.1% in screening of NPC.^193^

It has widely been proven that cf EBV DNA detection was important for NPC screening. Moreover, recent development on the potential clinical use of cf EBV DNA, for example, risk stratification, has also been proven. It was found that there is a correlation between pretreatment cf EBV DNA and NPC tumor stage and survival. Therefore, it has been recently confirmed that pretreatment cf EBV DNA could complement TNM staging for clinical prognostication as its ability to present key biological information reflecting tumor intrinsic aggressiveness that is not provided by TNM staging.^194^

Notably, detection of cf EBV DNA requires specialist equipment and expertise that is not available in the majority of hospitals. Therefore, a more inexpensive and simple detection technology of EBV DNA is needed.^195^ Recently, clustered regularly interspaced short palindromic repeats (CRISPR) technique has been used for pathogen detection. A platform called specific high‐sensitivity enzymatic reporter unlocking (SHERLOCK) combines Cas13a and recombinase polymerase amplification (RPA) to provide rapid and analytically sensitive detection for nucleic acid. Wu et al established this platform for plasma EBV DNA detection.^196^ They found that the EBV DNA concentration measured by SHERLOCK platform is comparable with that of QPCR with a Pearson correlation coefficient of *R* ^2^ = .9314, *P* < .0001. Moreover, the SHERLOCK platform can work at room temperature, which is a simpler, cheaper, and more convenient than the traditional QPCR method for EBV DNA detection.

Although it is widely accepted that plasma EBV DNA is a promising biomarker of precision screening for NPC, its level is lower in early‐stage NPC, making it hard to be reliably detected in these cancers. Scientists have tried to use NP brush samples for EBV DNA detection. Zheng et al collected NP brushing samples from healthy control, high‐risk control, and NPC patients.^197^ They found that EBV DNA detection from NP brush sample had a sensitivity rate of 96% and specificity rate of 97% when the cutoff value of 225 copy/ng DNA was used. Comparing to VCA‐IgA and plasma EBV DNA, EBV DNA from NP brushing samples demonstrated improved performance for NPC diagnosis. This suggests that the detection of EBV DNA in NP brush samples could be a more reliable method for NPC screening and diagnosis with the added bonus of being minimally invasive and low cost.

It is worth noting that the inconsistency of DNA levels among different laboratories is commonly known and effort is being made to address this issue. Recently, the standard for EBV DNA quantification was first developed by World Health Organization (WHO), which may be helpful for improving the comparability of EBV DNA results among different laboratories and assays.^198^

### 4.3 EBV‐encoded microRNAs (miRNAs)

EBV, the first virus reported to produce its own miRNAs,^199^ encodes BART miRNAs and BHRF1 miRNAs.^200^, ^201^, ^202^, ^203^ Multiple studies have confirmed that high expression of BART miRNAs and low level of BHRF1 miRNAs are detected in NPC tissues.^204^ Currently, there are 22 miRNA precursors encoded by the BART gene, of which 44 mature BART miRNAs are associated with the pathogenesis of NPC.^205^.Therefore, plasma circulating miRNA was supposed to be a potential biomarker for NPC diagnosis.^206^, ^207^, ^208^

Zheng et al compared the level of miRNAs between biopsies and NP brush samples from NPC and control groups by quantitative real‐time PCR (QRT‐PCR) and found that the expression of four BART miRNAs is higher in NPC patients.^209^ Among these miRNAs, miR‐BART1‐5p is the most promising biomarker for NPC diagnosis (93.5% sensitivity and 100% specificity). Moreover, miR‐BART1‐5p, when combined with VCA‐IgA, EA‐IgA, and plasma EBV DNA, was able to reduce the misdiagnosis rate of NPC.

Zhang et al compared the expression profiles of EBV BART miRNAs between EBV latently infected cell lines (Mutu I and Mutu III), EBV‐positive NPC cell line (C666‐1), and hTERT‐immortalized NP epithelial cell line (NP460‐hTERT‐EBV).^210^ They found that miR‐BART3, miR‐BART7, and miR‐BART13 are secreted at high levels from NPC cells. They then measured these three BART miRNAs expressions in the plasma samples of NPC patients, non‐NPC tumor patients, and healthy controls and found that only miR‐BART7 and miR‐BART13 are evaluated in NPC patients compared to the other two control groups. Combining with these two miRNAs showed an excellent predictive value for NPC diagnosis.

Recently, several researches have showed that EBV‐encoded miRNAs are released into the extracellular environment via exosomes by EBV‐infected cells. Ramayanti and colleagues^211^ found that the miRNAs secreted by C666‐1 are bound to extracellular vesicles (EVs). And miR‐BART13‐3p was further found to be higher in serum EVs of NPC compared to that of healthy controls. The authors suggested that circulating EV‐bound miR‐BART13‐3p should be taken into consideration for NPC screening.

The above studies demonstrate that EBV‐encoded miRNAs with the most diagnostic potential vary between different studies, possibly due to variation between samples.^212^ Therefore, further large‐scale studies should be conducted to estimate the diagnostic value of these miRNA.

### 4.4 Genetic susceptibility loci of NPC

To identify the possible susceptibility genes of NPC, a series of research methods, such as linkage studies, case‐control association studies, and genome‐wide association studies (GWAS), have been widely conducted.^213^, ^214^

As an important method of studying susceptibility genes, linkage analysis provided direct evidence of genetic susceptibility loci of NPC. In 2002, Feng et al conducted that a linkage study contained members of 20 high‐risk Cantonese‐speaking families. By using polymorphic microsatellite markers, which could cover 22 autosomes with an average density of 10 cM, they found that the major susceptibility locus of NPC is on chromosome 4p15.1‐q12.^215^ Other linkage studies have identified other susceptibility loci such as 6p21, 3p21.31−21.2, and 5p13 instead of 4p15.1−q12 in NPC families of Southern Chinese origins.^213^ These different results suggest that there is significant genetic heterogeneity in the occurrence of NPC.

Genes involved in the tumorigenesis, DNA damage and repair, immune regulation, and metabolism of carcinogens have been clarified for their relevance with NPC by case‐control studies.^216^ Jia et al found that multiple mutation sites on the cytochrome P4502E1 gene (CYP2E1) were significantly correlated with the incidence of NPC through a control study of NPC cases based on Guangdong population.^217^

In contrast, GWAS is a hypothesis‐independent approach, enabling us to test each polymorphic site of NPC susceptibility in a genome‐wide level. Bei et al conducted the largest scale sample size GWAS of NPC patients in Southern Chinese.^218^ They discovered three new and significant susceptibility loci, including TNFRSF19, MECOM, and CDKN2A‐CDKN2B gene at 13q12, 3q26, and 9p21, respectively. Nowadays, more and more susceptibility loci have been identified by GWAS, such as ITGA9 at 3p22.2, MICA at 6p21.33, HLA‐DQ/DR at 6p21.32, and so on.^213^

As more genetic susceptibility genes of NPC are discovered, scientists should be able to improve and develop the risk prediction model for high‐risk population screening, which could effectively reduce the incident and mortality rate of NPC.

### 4.5 EBV subtypes

Previously identified susceptibility genes and environmental factors can increase the risk of NPC but cannot fully explain for the high incidence of NPC in certain regions, suggesting that other risk factors have yet to be identified.^219^ There has been much speculation about the possible contribution of EBV strain variation to the development of NPC and other EBV‐associated cancers. Previous studies have identified strain variation over certain regions of the EBV genome (namely, the EBNA2 and EBNA3 regions) that have resulted in EBV isolates being classified as either EBV‐1 (like the prototype B95.8 virus) or EBV‐2 (like the Jijoye or AG876 virus). In addition to this broad distinction between EBV types 1 and 2, there is also minor heterogeneity within each virus type. Individual strains have been identified on the basis of changes, compared with B95.8, ranging from single base mutations to extensive deletions. As for functional differences between different EBV strains, one study showed that an EBV isolate cloned from NPC (M81) is more efficient at infecting epithelial cells and more lytic in B cells, supporting the possibility that virus strain variation contributes to the risk of developing NPC.^220^ Furthermore, a recent study has identified that the EBV‐encoded EBNA1 protein derived from NPC has different properties to the prototype B95.8 EBNA1 that may impact the carcinogenesis.^221^ A recent large‐scale study was conducted to explore the role of EBV strain variation in the development of NPC in Southern China. By using the whole‐genome sequencing, Xu and her colleagues analyzed 270 EBV isolates and compared the EBV genomic variation between patients with EBV‐related cancers and healthy controls.^222^ Moreover, they finally found two nonsynonymous variants of gene BALF2 (SNP 162476_C and SNP 163364_T), which indicated the high‐risk EBV subtypes for NPC in Southern China. This study supports the contention that there are high‐risk NPC variants of EBV and this provides opportunities for the development of novel therapeutic agents and diagnostic tests as well as more specific approaches for population screening and prophylactic vaccination.

## 5 EVOLUTION OF NPC TREATMENT

Primary treatment modalities for NPC include radiotherapy, chemotherapy, surgery, immunotherapy, and targeted therapy. The development of these therapies has improved the management and survival rates of NPC patients.^173^, ^223^, ^224^, ^225^, ^226^

### 5.1 Radiation therapy

Traditionally, radiation therapy is commonly used to treat NPC because the tumor is highly sensitive to ionizing radiation and the anatomical location where it occurs limits the surgical approach. Radiotherapy is generally effective for patients with early and nonmetastatic NPC; therefore, it is the main treatment for the nonmetastatic stages.^227^, ^228^

Among different radiotherapy approaches, intensity‐modulated radiotherapy (IMRT) is the most widely used treatment method today, and it has many advantages over conventional delivery approaches such as 2D‐RT (two‐dimensional radiation therapy) or 3D‐CRT (three‐dimensional conformal radiation therapy). IMRT sculpts the radiation doses to the shape of the specific areas within tumors to ensure good local control while minimizing side effects by using computer‐controlled linear accelerators.^229^

The improved efficacy of IMRT comparing to 2D‐RT was proven in a randomized phase III trial of 616 patients with localized/locoregional disease, where IMRT was found to yield better survival with less toxicity.^230^ A meta‐analysis including 3570 patients suggested that the IMRT group had better 5‐year local control (OR, 1.94 [1.53‐2.46]) and overall survival (OS) rate (1.51 [1.23‐1.87]) compared with the traditional radiotherapy groups (2D‐RT and 3D‐CRT), with substantially lower rate of late radiation toxicity, including trismus, xerostomia, and temporal lobe neuropathy.^231^

In addition to IMRT, new methods such as intensity‐modulated carbon ion therapy (IMCT) and intensity‐modulated proton therapy (IMPT) have also attracted interest. Comparative studies suggested that normal tissues can be better spared when using IMPT, while the effective delivery of target dose to the lesion is maintained.^232^, ^233^ Hu and colleagues showed that retreatment with carbon ion therapy in patients with locoregionally recurrent NPC yielded promising outcomes (1‐year OS rate 98.1%, local recurrence‐free survival rate 86.6%, and regional recurrence‐free survival rate 97.9%) with rare severe toxicities.^234^

Despite encouraging results from IMPT and IMCT, these different approaches have not been compared in prospective clinical trials with IMRT. Thus, further research is required to bring more information on the efficacy and safety toward these novel technologies. Tomotherapy is another radiation planning and delivery technology that may allow higher doses of radiation to be delivered with less damage to normal tissues. The difference of efficacy and life‐quality between tomotherapy and IMRT is under investigation in an ongoing clinical trial conducted among NPC patients (NCT03588403).

Considering the critical tissues adjacent to the tumor site, precise dose delivery to the target volumes and accurate delineation of organs at risk (OARs) are of great importance, but is challenging and highly variable among radiation oncologists.^235^ In an effort to decrease contouring variability, Lee et al published practical contouring guideline based on consensus opinions from international NPC experts.^236^ Lately, deep convolutional neural networks have been investigated to train an artificial intelligent (AI) contouring tool capable of generating gross tumor volume (GTV) contours. Compared with ground truth contours generated by human experts, the AI‐generated contours demonstrated improved accuracy.^237^

### 5.2 Chemotherapy

NPC is highly sensitive to chemotherapy, with a reported response rate up to 80% for cisplatin‐based chemotherapy.^238^, ^239^ Early‐stage NPC can be treated with radiotherapy alone. For intermediate and advanced‐stage NPC, chemotherapy is usually used previous or concurrent to radiotherapy or as an adjuvant after radiotherapy. Treatments for metastatic NPC are mainly composed of a combination of cytotoxic chemotherapy, including gemcitabine, 5‐FU, paclitaxel, and cisplatin.

Recent studies have shown that the 10‐year disease‐specific survival (DSS) rate of high‐risk stage III NPC patients receiving chemoradiotherapy is higher than that of patients receiving IMRT alone, while the difference between the survival rate of patients with stage II and low‐risk stage III disease is not detected.^240^ Currently, for stage II‐IVA NPC, induction or adjuvant chemotherapy followed by concurrent CRT is considered by the NCCN (National Comprehensive Cancer Network) Guidelines as level 2A evidence for clinical recommendation. For patients with oligometastatic NPC, approaches such as radical CRT to the primary tumor and ablation of the oligometastases can render good long‐term prognosis in some patients.^241^, ^242^, ^243^ Among the different chemotherapeutic drugs, platinum‐based regimen, with concurrent cisplatin followed by adjuvant cisplantin/5‐FU or induction gemcitabine/cisplatin followed by concurrent cisplatin, is generally preferred for patients with locoregionally advanced NPC.^242^ In a phase III, randomized multicenter trial involving 480 patients, gemcitabine, and cisplatin as induction chemotherapy combined with concurrent CRT was compared with concurrent CRT alone. The addition of induction chemotherapy significantly improved recurrence‐free survival (RFS) and OS rate comparing to RT alone (hazard ratio [HR] for RFS: 0.51; 95% [CI], 0.34 to 0.77; HR for OS 0.43; 95% [CI], 0.24 to 0.77). Thus, induction chemotherapy plus concurrent CRT is a more promising treatment option than CRT alone.^244^

### 5.3 Surgery

Surgery is not often used as a first‐line treatment for NPC because the nasopharynx is deeply situated adjacent to key neurovascular structures. However, for isolated neck recurrent lesion after RT or CRT or residual nodal disease, neck dissection can be used. Nasopharyngectomy is more commonly used in Asia as a treatment option for small localized recurrences.^245^ Traditional surgical approaches were associated with significant complications and low rate of complete resection, thereby limiting its application.

In recent years, minimally invasive technologies such as endoscopic nasopharyngectomy have been successfully applied to the resection of early‐stage recurrent NPC with relatively low medical costs.^246^, ^247^ With continuing improvement in endoscopic technologies and accumulating surgical experience, endoscopic nasopharyngectomy can potentially overcome the shortcomings of traditional surgery and be applied to tumors other than small NPC recurrences.^248^ Some doctors believe that endoscopic techniques can be an alternative strategy for staged I NPC patients, and a recent study that included 10 stage 1 patients demonstrated that endoscopic nasopharyngectomy alone was associated with better treatment outcomes, lower medical costs, and higher quality of life (QOL), as compared with salvage IMRT.^249^ A nonrandomized prospective trial enrolling 189 staged I NPC patients focusing on evaluating the life quality and survival outcomes of patients treated with endoscopic nasopharyngectomy compared to IMRT is in progress (NCT03353467). This study will further verify these results. However, a randomized controlled trial is need to compare surgery to reirradiation ± systemic therapy in recurrent NPC patient to address confounding factors, such as tumor size, skull base involvement, patient performance status, etc.^250^

In general, advances in surgical and radiation technology (such as particle beam therapy) will provide patients with more treatment options, which is expected to improve the survival and quality of life of patients with locally recurrent or early‐stage NPC.^173^

### 5.4 Immunotherapy

Current immunotherapeutic approaches can be divided into three categories: immune checkpoint inhibition (ICI), adoptive immunotherapy, and active immunotherapy.

#### 5.4.1 Immune checkpoint inhibition

ICI is based on the principle, assuming that the body has sufficient adaptive immune response capabilities, but is inhibited by immune evasion mechanisms of tumor cells, and therefore, this approach aims to enhance the preexisting immune effect.^251^, ^252^, ^253^, ^254^ Programmed cell death protein 1 (PD‐1) is a cell surface receptor that broadly expressed on the T‐cells.^255^, ^256^ When bound to PD‐L1 on the surface of cancer cells, it activates inhibitory signals on T cells, thereby blocking the antitumor effect of T cells and leading to T cell exhaustion, which represents a state of progressive effector function losing. Similarly, CTLA‐4 also acts as a brake and sent negative signals hampering T‐cell activation.^257^ Under inhibitory effect of PD‐1, the cytotoxicity of CTLs to lyse tumor cells through granzyme B and perforin is limited, and the production cytokines such as TNF‐α, IL‐2, and IFN‐γ is reduced, creating an immunosuppressive microenvironment, which facilitates tumor persistence and progression.^258^, ^259^

The expression of PD‐L1 has been noticed in most NPC tumors, and high PD‐L1 expression has been linked to some extent to a high risk of recurrence and metastasis after conventional therapies, leading to poor clinical prognosis.^260^, ^261^, ^262^ Blocking the PD‐1/PD‐L1 pathway can stimulate anergic CD8+ T cells and restore their normal function such as proliferation, cytokines secretion, and cytotoxicity toward malignant cells. This may be a promising new therapy for NPC patients where coexpression of PD‐1/PD‐L1 is identified in the tumor biopsy (Table 2).

**TABLE 2:** Ongoing clinical trials of PD‐1 inhibitors or anti‐PD‐1 monoclonal antibodies for NPC

Experimental regimen	Control regimen	Sample size	Clinical setting	Phase	NCT number	Estimated completion date
Camrelizumab+Gemcitabine+Cisplatin	Placebos+Gemcitabine+Cisplatin	250	Recurrent/metastatic NPC	III	NCT03707509	August 2021
Camrelizumab	Observation	400	Stage III‐IVA NPC	III	NCT03427827	February 2022
Pembrolizumab	Capecitabine/gemcitabine/docetaxel	233	Recurrent/metastatic NPC	III	NCT02611960	November 2020
Sintilimab+Gemcitabine+Cisplatin+IMRT	Gemcitabine+Cisplatin+IMRT	420	Stage III‐IVA NPC	III	NCT03700476	January 2023
Tislelizumab+Gemcitabine+Cisplatin	Placebo+Gemcitabine+Cisplatin	256	Recurrent/metastatic NPC	III	NCT03924986	August 2021
Toripalimab+gemcitabine+cisplatin	Placebo+gemcitabine+cisplatin	280	Recurrent/metastatic NPC	III	NCT03581786	August 2020
AK105		153	Stage IVb NPC	II	NCT03866967	June 2020
PDR001	Chemotherapy	122	Recurrent/metastatic NPC	II	NCT02605967	August 2020

Pembrolizumab and nivolumab, both PD‐1 blocking antibodies, have been approved by the FDA for recurrent or metastatic head and neck squamous cell carcinoma (HNSCC), and their effectiveness and safety was proved in single arm phase I/II trial in NPC.^263^, ^264^ Similarly, a phase II trial of nivolumab in 44 patients with multiply pretreated recurrent/metastatic NPC showed a response rate of ∼20% with no unexpected toxicity.^264^ These promising results have led to many large randomized trials testing the effectiveness of PD‐1 immune checkpoint therapy against conventional chemotherapy in recurrent/metastatic NPC patients, including those testing camrelizumab (NCT03707509, NCT03427827), pembrolizumab (NCT02611960), sintilimab (NCT03700476), toripalimab (NCT03581786), tislelizumab (NCT03924986), and AK105, another monoclonal antibody directed against PD‐1 (NCT03866967).

Although effective as monotherapy, there is an increasing evidence that chemotherapy and/or radiation may be used in conjunction with ICI to augment its efficacy and produce more durable clinical responses in different solid cancers, including NPC. Some studies suggest that after RT and chemotherapy, PD‐L1 is upregulated, which promotes T cell apoptosis and limits the immune response. The participation of anti‐PD1 or anti‐PD‐L1 agents would prevent this effect, thereby promoting immunity against tumors.^265^, ^266^ As an emerging treatment alternative, ICI is also being studied in earlier settings for NPC, including first‐line recurrent/metastatic or curable settings. However, the most optimal combinations for effective responses, the additional benefit of immune adjuvant drugs, and the optimal sequence for radiotherapy and chemotherapy have yet to be determined.^267^

#### 5.4.2 Adoptive immunotherapy

The second approach is adoptive immunotherapy that bypasses the antigen presentation step and involves the infusion of directly activated effector cells, such as natural killer (NK) cells and CD8+ cytotoxic T lymphocytes (CTLs).^268^

NK cells are lymphocytes of the innate immune system, which can induce tumor cell death through perforin‐/granzyme‐mediated cytotoxicity.^269^ Tumor or virus‐infected cells can be recognized by NK cells via a variety of mechanisms.^270^, ^271^, ^272^ In addition, NK cells affect the development of antigen‐specific T cell responses through interaction with dendritic cells (DCs) and the secretion of IFNγ. Therefore, direct cytotoxic effects of NK cells on cancer cells in adoptive immunotherapy can inhibit tumor development and further stimulate downstream adaptive antitumor immune responses against cancer.^273^, ^274^ There have been two clinical study registered infusing autologous NK cells in efforts of treating NPC. The first one is a pilot clinical trial to study the feasibility and safety of this immune therapy including collecting, manipulating, and infusing autologous‐enriched NK cells. NK cells were selected and activated by short‐term incubation in IL‐2 and reinfused into patient with metastatic NPC. To assess immune response, EBV‐specific T cells, quantitation of regulatory T cells, NK cell function, and serum cytokine levels were monitored postinfusion (NCT00717184). Another phase I/II study combined cetuximab (an anti‐EGFR monoclonal antibody) in order to direct NK cells to the target site, provided that the epidermal growth factor receptor (EGFR) is ubiquitously expressed in HNSCC as well as NPC. In this study, expanded autologous NK cell was infused with cetuximab to patients with refractory NPC and HNSCC (NCT02507154). However, both aforementioned studies did not provide strong evidence, suggesting that the NPC patients could benefit from autologous NK cell infusion as main therapy.

Of the poorly immunogenic latent EBV antigens that are expressed in NPC, EBNA1 is more frequently expressed in host cells and can be recognized by CD4+ T cells as a specific antigen, while CTLs recognize epitopes of LMP1 and LMP2 presented by the infected cells.^275^

In this approach, cytotoxic cells, such as tumor infiltrating T lymphocytes and/or NK cells, are taken from the patients, expanded in vitro, and then transferred back to the same patients. EBV‐specific CTLs generated in vitro from the patient's own blood or from HLA‐matched healthy donors are transferred to the patients hopefully resulting in homing to the tumor site, recognition of the processed virus antigens on the NPC cells, and cytotoxicity via perforin‐induced apoptosis. LMP2 is more consistently expressed in NPC and regarded as a more potent latent‐phase immunogen, compared to LMP1, to be applied in CTLs‐based immunotherapy.^275^ Adoptive transfer of EBV‐specific CTLs with or without systemic lymphodepletion has been tested in NPC in several clinical trials (Table 3).

**TABLE 3:** Ongoing clinical trials of adoptive immunotherapy for NPC

Experimental regimen	Intervention model	Sample size	Clinical setting	Phase	NCT number	Estimated completion date
Tumor‐infiltrating lymphocytes (TILs)	Parallel, Randomized	116	Locoregionally advanced NPC	II	NCT02421640	Mar 2020
EBV‐specific CTL	Parallel, Randomized	330	Recurrent/metastatic EBV‐positive NPC	III	NCT02578641	Jan 2023
Epcam CAR‐T cells	Single Group	30	Recurrent/refractory NPC	I	NCT02915445	Jul 2022
LMP2 antigen‐specific TCR‐T cells	Single Group	27	Recurrent/metastatic NPC	I	NCT03925896	Aug 2022
PD‐1 knockout EBV‐CTL	Single Group	20	Stage IV EBV positive malignancies	II	NCT03044743	Mar 2022
TGF‐beta‐resistant cytotoxic T cells	Single Group	14	Recurrent/refractory EBV positive NPC	I	NCT02065362	Feb 2033
LMP, BARF‐1, and EBNA1‐specific CTLs (MABEL CTLs)	Parallel, Nonrandomized	42	EBV positive tumor	I	NCT02287311	Mar 2025

In 2001, the first pilot study evaluating the clinical effect as well as safety profile of adoptive cellular therapy included 4 NPC patients and it showed that the treatment was safe, did not cause inflammation or other complications, and was associated with significant antiviral responses as evidenced by decreased plasma EBV DNA levels and increased CTL levels.^276^ Comoli and colleagues conducted a phase I clinical trial involving 10 refractory or metastatic NPC patients, and showed that the adoptive T‐cell therapy was well tolerated by patients, with a temporary stabilization of disease. In addition, a significant expansion of endogenous tumor‐infiltrating CTLs was induced after treatment, as well as a long‐term increase in LMP2‐specific immunity.^277^ The addition of EBV‐CTL following standard first‐line chemotherapy in recurrent/metastatic NPC patients has also been explored. Additionally, adoptive EBV‐CTLs transfer combined with gemcitabine and carboplatin (GC) chemotherapy was delivered in a phase II trial including 38 advanced EBV‐positive NPC patients. The treatment was well tolerated in 35 patients who received CTLs after chemotherapy, and the ORR and clinical benefit rate were 42.9% and 62.9%, respectively.^278^

Tumor‐infiltrating lymphocytes (TILs) contain a high number of EBV‐specific T cells that are likely to be more effective at infiltrating into the tumor microenvironment. Therefore, obtaining those autologous TILs from tumor site and transferring back to patient after in vitro proliferation has been proposed as an effective strategy. A study involving 23 patients with locoregionally advanced NPC showed that 19 of 20 patients who received both CRT and TILs infusion achieved an objective antitumor response with no unexpected side effects.^279^ The effectiveness TILs plus CRT versus CRT alone is being tested in a phase II trial in patients with locoregionally advanced NPC (NCT02421640).

Recently, in a phase I/II study, the effectiveness of EBV‐specific CTLs immunotherapy was investigated in 28 recurrent/metastatic NPC patients (21 were treated). Despite a complete and durable response (remission period >8 years) to this therapy was reported in one case, the ORR was low (1 complete response in 21 patients). It is worth mentioning that despite failing EBV‐CTL immunotherapy, some tumors responded to the same chemotherapy regimen (docetaxel, gemcitabine) to which they were previously resistant. This suggests that modification of the immune system with immunotherapy may allow patients to have a renewed response to prior chemotherapy regimens.^280^ The aforementioned studies are mainly performed in small cohorts of patients, and more definitive data will come from ongoing clinical trials in larger patient populations that are exploring whether this type of immunotherapy can be combined into the standard treatment of NPC. A multicenter, randomized phase III trial is comparing GC followed by adoptive EBV‐specific autologous CTL therapy to the same chemotherapy regimen alone and is enrolling 330 recurrent/metastatic NPC patients from 30 centers across Asia and the United States (NCT02578641), which may provide a new and better treatment for NPC patients.

Lately, engineered T‐cells have emerging as another promising strategy to improve the clinical success of adoptive immunotherapy, such as the use of chimeric antigen receptors (CARs)‐engineered T‐cells or modified T‐cells with genetically modified T cell receptors (TCRs). The general process of this treatment is to add chimeric tumor‐recognizing antibodies to the surface of patient‐derived T‐cells by using genetic engineering technology, and transfer these cells back into the patient after ex vivo expansion.^281^

An ongoing clinical study (NCT02915445) aims to determine the safety of CAR‐T cells designed to recognize the epithelial cell adhesion molecule (EpCAM) in NPC or breast cancer patients. Chen and colleagues constructed LMP2A CAR‐T cells and showed targeting cytotoxicity in LMP2A‐positive NPC cells. A single‐arm, phase I clinical trial has been activated and it will estimate the effectiveness and safety profile of LMP2‐specific TCR‐T cells as an adoptive immunotherapy strategy for recurrent/metastatic NPC in China (NCT03925896).^250^ The therapeutic effect of MABEL CTLs, T cells stimulated by LMP, BARF‐1, and EBNA1 in vivo, is also being examined in patients with EBV‐positive malignancies (NCT02287311). Moreover, modified T cells, such as dominant negative receptor (DNR) T cells loaded with the DNR and PD‐1 knockout CTLs, are under investigation in two phase I clinical trials (NCT02065362, NCT03044743).

#### 5.4.3 Active immunotherapy

The third strategy includes active immunotherapy approaches, which aim to enhance the tumor‐specific cellular immunity by delivering tumor antigen‐loaded antigen presenting cells (APCs), modified vaccinia Ankara virus (MVA), or peptide‐based vaccination. Some studies have demonstrated that these approaches stimulate a moderate antitumor effect in a subset of NPC patients.^282^, ^283^, ^284^, ^285^

DCs, known as professional antigen‐presenting cells, are critical for the adaptive immune response initiation and are being studied as a therapeutic strategy to treat metastatic NPC. Monocyte‐derived DCs (moDCs) from metastatic NPC patients were cultured ex vivo and injected into lymph nodes in the inguinal region of the patients with EBV LMP2 epitope‐containing polypeptides. In 9 of 16 NPC patients, a boosted cytotoxic T‐cell immune response was noticed. And a partial response was obtained in two patients.^286^

Moreover, there are other approaches that can strengthen DC's antigen presentation and subsequent CD4+ and CD8+ T cells response. For example, using adenovirus encoding antigenic peptides as vectors to infect DCs in vitro.^287^ In a phase II clinical trial, 16 patients with metastatic NPC were vaccinated with autologous DCs infected with Ad‐ΔLMP1‐LMP2 vaccine, composed of adenovirus encoding full‐length LMP2 and a truncated version of LMP1 (ΔLMP1). The results revealed that the Ad‐ΔLMP1‐LMP2 vaccine was well tolerated, but there was no expansion of peripheral LMP1/2‐specific T cells, and only limited efficacy was observed. The authors suggested that future studies should focus on administering a more potent DC vaccine to patients with lower tumor burden.^288^ CD137L‐DC is a novel monocyte‐derived inflammatory DC (moDCs) induced by agonists of CD137 ligand, which is expressed as a transmembrane protein on the surface of DCs, NK cells, and other types of leukocytes. The investigators suggest that the CD137L‐DC will enhance the proliferation of type 1 T helper (Th1) cells in response to tumor‐associated antigens and activate a more powerful type 1 CD8+ T cells response than the commonly used moDCs. The safety and recommended dose of an EBV‐specific CD137L‐DC vaccine (CD137‐DV‐EBV‐VAX) will be investigated in a phase I clinical trial involving recurrent/metastatic or locally advanced NPC patients (NCT03282617).^282^ The EBV antigens expressed in NPC can also be designed as peptide vaccines and either used directly as vaccines with an appropriate adjuvant or pulsed onto autologous DCs.

In addition, other immune cells such as cytokine‐induced killer (CIK) cells, produced from PBMC incubation with cytokines ex vivo, represent a group of mixed immune effector cells that have antitumor effect. One randomized trial compared the efficacy of autologous CIK transfusion in combination with GC chemotherapy versus GC chemotherapy alone in 60 metastatic NPC patients. The GC+CIK group showed an expressively longer progression‐free survival (PFS) than the GC alone group.^289^ Another ongoing phase II trial investigates the efficacy of concurrent chemotherapy with CIK cells and DCs (DC‐CIK) versus chemotherapy alone in patients with treatment‐refractory solid tumors including NPC (NCT03047525). Larger validation studies are needed to determine the efficacy of this novel immunotherapy approach.

Some of the EBV antigens that can be detected in host cells may also be displayed in peptide vaccines as potential immunogenic epitopes. By expressing viral latency antigens, the vaccine aims to activate T‐cells immune response and exploits their antitumor cytotoxicity by presenting these antigen epitopes on their surface.

A therapeutic MVA virus vaccine comprising a recombinant vaccinia Ankara vector encoding an EBNA1/LMP2 fusion protein (MVA‐EBNA1/LMP2) so that the fusion protein can be expressed endogenously and efficiently processed via both HLA classes I and II. This approach was found to efficiently reactivate EBNA1‐ and LMP2‐specific CD4+ and CD8+ T‐cell responses in vitro.^290^ The vaccine was then tested in two clinical trials (in the United Kingdom and Hong Kong, separately) with results showing that the MVA‐EBNA1/LMP2 vaccine is well tolerated and can induced EBNA1‐ and LMP2‐specific T‐cell response across diverse ethnicities.^285^, ^291^ Whether the vaccine can enhance EBV‐specific responses in a wider population of NPC patients and show early signs of therapeutic benefit is currently being examined in a phase II study. Lutzky et al generated an adenovirus‐based vaccine by constructing a replication‐deficient adenovirus vector pulsed with LMP1, LMP2, and EBNA1 peptide (Ad‐SAVINE) (NCT01094405). Zeng and colleagues used a recombinant replication‐deficient adenovirus type 5 vector encoding the full‐length LMP2 as the rAd‐LMP2 vaccine that can induce LMP2‐specific cytotoxicity against LMP2‐expressing tumor cells in vitro.^292^ Rühl et al have created a vaccination strategy by using either EBNA1‐specific DCs or adenovirus combined with modified vaccinia virus Ankara (MVA) booster, named heterologous prime‐boost strategy. This strategy stimulated effective T‐cell‐mediated and humoral immune response in mouse models, suggesting that it could have a role in the clinical management of NPC.^281^, ^293^ However, despite the ability of these different adenovirus vaccine/prime‐boost combinations to activate T‐cell, their safety and efficacy in NPC patient need to be evaluated in patients.

### 5.5 Molecular‐targeted therapies

To date, only two pathways have been widely studied for targeted therapy in NPC; they are the VEGF receptor (VEGFR) and the EGFR pathways. The clinical data for targeting these pathways are summarized below.

#### 5.5.1 Vascular endothelial growth factor receptor

It has been shown that high expression of VEGF in NPC is associated with unsatisfactory prognosis.^294^, ^295^, ^296^, ^297^ The activation of VEGF signaling cascade plays an important role in promoting tumor growth, angiogenesis, and metastasis, thereby providing a promising therapeutic target.^175^ Agents targeting the VEGF that has demonstrated encouraging activities in clinical trials include a group of tyrosine kinase inhibitors (TKIs) of VEGFR, such as sorafenib, sunitinib, axitinib, famitinib, and pazopanib (Table 4).

**TABLE 4:** Selected clinical trials of molecular‐targeted therapies for NPC

Experimental regimen	Phase	Size	Clinical setting	ORR	Median PSF (mon)	Median OS (mon)	Additional results	Reference
Sorafenib	II	54	R/M NPC	77.80%	7.2	11.8	NR	Xue et al^299^
Axitinib	II	40	R/M NPC	NR	5.0	10.4	1‐year OS: 46.3%	Hui et al^302^
Endostatin	II	72	Metastatic NPC	77.80%	12	19.5	1‐year PFS: 45.4%; 1‐year OS: 87.4%	Jin et al^305^
Cetuximab	II	60	R/M NPC	11.70%	2.7	7.7	CBR 60%; PR: 11.7%; SD: 48.3%; PD:38.3%	Chan et al^312^
Cetuximab (vs cisplatin)	II,RCT	44	Stage III‐IVb NPC	100% (both)	NR	NR	3‐year DFS: 78.3%(vs 85.7%, *P* = .547); 3‐year OS :100% (vs 95.70%, *P* = .619)	Xu et al^314^
Nimotuzumab	Retro	210	Nonmetastatic NPC	100%	NR	NR	5‐year PFS: 91.7%; 5‐year OS: 88.7%	Wang et al^318^
Nimotuzumab	Retro	42	Locally advanced NPC	100%	NR	NR	2‐year LRFS: 96.4%; DMFS: 93.1%; OS: 96.6%	Liu et al^319^
Nimotuzumab (vs docetaxel)	RCT	118	Stage III‐IVa NPC	70.6% (vs 61.7%)	NR	NR	Cervical lymph nodes response rate: 81% (vs 60%, *P* = .036); nasopharyngeal lesions response rate: 60.3% (vs 51.7%, *P* = .446)	Lu et al^320^
Nimotuzumab or cetuximab	Retro	203	R/M NPC	67.50%	8.9	29.1	1‐year PFS: 35.5%; 1‐year OS: 42.5%	Chen et al^227^

Abbreviations: ORR, overall response rate = CR + PR; CBR, clinical benefit rate = CR + PR + SD; CR, complete response; DFS, disease‐free survival; DMFS, distant metastasis‐free survival; LRFS, local recurrence‐free survival; NR, not reported; OS, overall survival; PD, progression disease; PFS, progression‐free survival; PR, partial response; RCT, randomized controlled trial; Retro, retrospective analysis; R/M NPC, recurrent or metastatic nasopharyngeal carcinoma; SD, stable disease.

In a phase II clinical study focusing on head and neck cancer that enrolled seven NPC patients, sorafenib was well tolerated and showed a trend of longer time to progression, but this was not statistically significant.^298^ The combination of sorafenib, cisplatin, and 5‐fluorouracil was shown to be tolerable in 54 recurrent/metastatic NPC patients, but the contribution of sorafenib to the chemotherapy was not clear.^299^ In a phase II trial of pazopanib in 33 patients with recurrent/metastatic NPC, the partial response rate was 6.1%, with the clinical benefit rate of 54.5%.^300^

Sunitinib was assessed for its efficacy and safety in 14 pretreated recurrent/metastatic NPC patients. This study demonstrated modest clinical activity, but among patients previously receiving high‐dose RT, high incidence of upper gastrointestinal bleeding was noticed.^301^

Axitinib was assessed in 37 heavily pretreated NPC patients in a phase II trial and showed a favorable safety profile, durable disease control, and a clinical benefit rate of 78% at 3 months and 43% at 6 months.^302^ In 2013, Huang et al investigated famitinib in 58 recurrent/metastatic NPC patients from eight sites in China in a phase II trial; the median PFS was 3.2 months and the clinical benefit rate was 33%, including five patients with a partial response and 16 with stable disease (NCT01392235).

Endostatin is an endogenous antiangiogenic inhibitor that prevents the angiogenic effects of growth factors, particularly VEGF.^303^ In 2005, the State Food and Drug Administration (SFDA) approved Endostar, a recombinant human endostatin drug, for advanced NSCLC (nonsmall cell lung cancer). The efficacy endostar in combination with GC chemotherapy was investigated in a phase II trial in 28 metastatic NPC patients.^304^ The median PFS was 19.4 months, and the OS rate and PFS rate was 90.2% and 69.8% at one year, respectively. Complete and partial response occurred in 14 and 10 patients, respectively. The investigators enrolled 44 more patients in additional to the previous 28 patients and reported their updated results in 2018. The ORR for the entire cohort was 77.8% (56 of 72), including 40 (55.6%) partial and 16 (22.2%) complete responders. The investigators concluded that the regimen of endostatin and GC chemotherapy was well tolerated and potentially highly effective in metastatic NPC.^305^ There is an ongoing phase II clinical study testing whether endostar plus IMRT can reduce the rate of severe toxicities for locally recurrent NPC patients when compared to IMRT alone (NCT02636231). Another study is in progress to determine the safety profile and efficacy of endostar coupled with nedaplatin and low‐dose 5‐FU compared with the same chemotherapy regimen alone in refractory NPC (NCT02590133).

#### 5.5.2 Epidermal growth factor receptor

EGFR, with its intrinsic tyrosine kinase activity, can bind to ligands of the EGF family and participate in controlling various cellular activities, such as proliferation, apoptosis, and differentiation. In many epithelial tumors, the EGFR signaling pathway is abnormally activated.^306^ It is worth noting that the overexpression of EGFR was reported in more than 90% of undifferentiated nonkeratinizing NPC, which is related to unsatisfactory porgnosis.^307^, ^308^, ^309^ The relatively high prevalence of positive staining for EGFR suggests that blockade of this pathway by either small molecule inhibitors or antibodies can provide a significant therapeutic benefit to advanced NPC patients.^310^, ^311^ Of existing EGFR inhibitors, cetuximab, gefitinib, nimotuzumab, and erlotinib have been studied clinically in NPC.

The efficacy and safety of combining cetuximab with carboplatin was assessed in a multicenter phase II clinical study involving 60 NPC patients who had received platinum‐based chemotherapy. The ORR was 11.7% [4.8%‐ 22.6%] and the OS time and time to progression are 8.3 and 2.9 months, respectively. A phase II clinical study including 30 patients, the effectiveness, and safety profile of cetuximab plus CRT was assessed, and there were 21 (70%) achieved clinical responses including 18 partial responses and 3 complete responses. The median OS, TTP, and 2‐year OS were 23.6, 12.2 months, and 53.3%, respectively. These studies concluded that cetuximab in combination with carboplatin or chemoradiation may provide clinical benefit and can be well tolerated in NPC patients.^312^, ^313^ Xu and colleagues compared the efficacy of cetuximab and cisplatin in a phase II RCT trial involving 44 stage III‐IVb NPC patients, and the two agents showed comparable effect with 3‐year disease‐free survival of 78.3% and 85.7% (*P* = .547), respectively.^314^ However, results with gefitinib and erlotinib are far from acceptable. Gefitinib demonstrated poor clinical response in two phase II trials, which used gefitinib in recurrent/metastatic NPC.^315^, ^316^ Similarly, erlotinib showed a poor response rate when used as maintenance therapy for recurrent/metastatic NPC patient after treated with standard gemcitabine‐platinum chemotherapy.^317^

Nimotuzumab (NTZ) was found to be a highly selective humanized monoclonal antibody with less toxicity (such as skin rash and mucosal reaction) as compared with cetuximab. There are several clinical trials assessing the use of NTZ in recent years. For locally advanced NPC, retrospective analysis suggested that NTZ combined neoadjuvant chemotherapy followed by concurrent CRT is effective and well tolerated.^318^ In one trial, 42 patients with locally advanced NPC were treated with NTZ combined with concurrent CRT. Moreover, the complete response and partial response was observed in 38 (90.5%) and 4 (9.5%) patients, respectively. The ORR reached 100% and the 2‐year OS is 96.6%, with no progressive disease observed at the initial evaluation.^319^ A multicenter randomized in 118 patients with stage III‐IVa NPC, induction nimotuzumab combined, and cisplatin/5‐FU (NPF group) was compared to induction docetaxel and cisplatin/5‐FU (DPF group); both arms were followed by concurrent CRT. The NPF group showed a higher ORR (81% vs 60%), more pronounced nodal response rate (*P* = .036), better tolerance to the following concurrent CRT, and milder adverse effect compared with the DPF group. Long‐term follow‐up evaluation is needed to determine whether this improvement is durable.^320^

As for the use of nimotuzumab in recurrent/metastatic settings, Zhao et al assessed the potential effect of nimotuzumab plus cisplatin/5FU as the first‐line chemotherapy after RT in the management of recurrent/metastatic NPC. The ORR was 71.4% (25/35), and the median PFS and OS were 7.0 and 16.3 months, respectively.^321^ Recently, a retrospective study of 203 R/M patients showed that anti‐EGFR antibody (CTX or NTZ) plus chemotherapy can achieve promising antitumor activity with a tolerable toxicity profile. The median PFS and OS were 8.9 months (95% CI: 7.7‐10.0) and 29.1 months (95% CI: 23.5‐34.6), respectively. In a multivariate analysis, the anti‐EGFR agent was an independent prognostic factors for PFS (*P* = .010). However, the benefit of anti‐EGFR antibody still warrants further evaluation in larger randomized controlled trails.^227^ An ongoing phase III trial is evaluating the efficacy of nimotuzumab versus cisplatin combined with IMRT in patients with stage II‐III NPC (NCT03837808).

Although the aforementioned studies have suggested that agents targeting VEGFR and EGFR pathways can provide clinical benefit for patients with advanced or metastasis NPC, but due to the limited number of patients participating in the experiments, we cannot fully weigh the benefit of the clinical application of targeted therapy for advanced NPC patients and further evaluation of combination therapy should be considered. Besides, there are many types of genetic mutations involved in NPC, including chromatin modification, cell cycle transformation, and phagocytosis, but unlike lung adenocarcinoma and colorectal adenocarcinoma, none of them in NPC are very frequent and common.^20^ And most of these mutations are without targeted drugs, which lead to limitation for targeted therapy of NPC. Recently, some inhibitors targeting EBNA1 showed promising properties and applications for treatment of NPC, which indicated that strategies targeting EBV will be of great value for targeted therapy of NPC.^322^

#### 5.5.3 Epigenetics regulators

Epigenetics is currently known to consist of three main components, which are heritable and affect gene expression, but do not alter DNA sequences. These mainly include DNA methylation, histone modifications, and noncoding RNA.^323^ These epigenetic processes are part of the normal machinery used by mammalian cells to regulate gene expression.^324^ However, some changes in epigenetic modifications are closely related to tumorigenesis.^325^

DNA methylation, commonly involved in gene silencing such as X‐chromosome inactivation and gene imprinting, are mediated by a variety of DNMTs. Methylation of a promoter CpG island influences the relationships between DNA and regulatory proteins, and thereby changes the transcription and expression of downstream genes.^326^ In NPC, various tumor suppressor genes involved in DNA repair, cell proliferation, apoptosis, cell adhesion, and migration were found to be downregulated by promoter methylation, suggesting that the aberrant methylation may play a role in NPC carcinogenesis.^327^ For example, LMP1 can hypermethylate and silence the E‐cadherin gene by activating DNMT1, thereby enhancing the invasiveness and migratory ability of NPC cells.^74^ Besides, there are several key genes in important signaling pathways related to NPC‐associated tumorigenesis, including p53, Wnt/β‐catenin, and Ras and Rho GTPase that are frequently disturbed by CpG methylation.^328^

Histone modifications, including acetylation, methylation, ubiquitination, sulfonylation, and phosphorylation, are also involved in the regulation of the expression of both EBV virus and host cell genes in EBV‐associated tumors. For example, the transcriptional repression of LMP1, EBNA2, EBNA3C, and BZLF1 by histone deacetylation suggests that histone deacetylases (HDACs) are involved in EBV latency maintenance and EBV‐induced tumorigenesis.^329^, ^330^

The close relationships of the epigenetic changes and tumorigenesis, as well as their plasticity and reversibility, make them become ideal potential drug targets for anticancer treatment, known as epigenetic therapies.^331^ Currently, hypomethylating agents and HDAC inhibitors are the two FDA‐approved classes of epigenetic drugs. Hypomethylating agents include drugs that inhibit DNMTs such as 5‐azacytidine (Vidaza) and decitabine, while HDAC inhibitors mainly consist of vorinostat, abexinostat, butyric acid, trichostatin A (TSA), romidepsin, and panobinostat.^330^, ^332^

By reactivating methylation‐silenced genes in EBV‐associated tumors, 5‐Azacytidine could enhance antitumor effect of immune system and therefore emerge as a potential approach to treat NPC.^239^ CC‐486, an oral formulation of azacitidine, was evaluated in a phase I clinical trial and resulted in three partial responses and four stable disease in eight NPC patients.^333^ However, the phase II clinical trial of CC‐486, involving 36 locally advanced or metastatic NPC patients, showed no significant clinical benefit with an ORR of 12% and a median PFS of 4.7 months.^334^ The clinical efficacy of decitabine is under investigation in a phase I/II clinical trial in which 30 NPC patients are being treated with decitabine combined with cisplatin‐induced chemotherapy followed by concurrent chemoradiotherapy (NCT03701451).

Histone modifications are mediated by enzymes, such as histone acetyl transferases (HATs) and HDACs. Since histone modifications are associated with maintaining the latent status of EBV, the inhibitors of HDACs provide a potential therapeutic strategy. In these approaches, reactivation of EBV results in the induction of lytic cycle proteins including virus‐encoded thymidine kinase and protein kinases that can activate acyclovir or related drugs thereby inhibiting DNA replication.^335^ Vorinostat, also known as SAHA, is a member of HDAC inhibitors that has been shown to induce EBV reactivation and lytic protein expression and could have therapeutic potential in NPC.^336^ A phase I clinical trial is evaluating the effect of the combination of vorinostat and azacitidine in recurrent of metastatic NPC patients without reported results (NCT00336063). Abexinostat, when combined with irradiation or cisplatin, showed increased antitumor effects in patient‐derived xenografts (PDXs) from metastatic NPC, suggesting that Abexinostat could potentially reduce treatment resistance.^325^, ^337^ Moreover, some studies suggested that inhibitors of DNMTs and HDACs, by enhancing tumor‐associated antigen expression, could reverse immune escape and improve the effectiveness of immunotherapy.^338^ The combination of epigenetic drugs and immunotherapy is being evaluated in different cancer types including solid tumor and hematological malignancies.^339^ However, we should keep in mind that the induction of EBV lytic cycle will induce the replication and assembly of EBV, thereby lysing host cells and exposing neighboring cells to more infectious EBV particles. Also, in recent years, it has been reported that the EBV lytic protein may contribute to carcinogenesis and several lytic proteins have homology with various antiapoptotic proteins.^340^, ^341^ They may also target tumor suppressors or further enhance genetic instability, leading to other pathological changes.^342^ Nevertheless, whether combining inhibitors of DNMTs or HDACs with chemoradiotherapy or immunotherapy could bring additional clinical benefit and the appropriate role of epigenetic therapies in NPC management still needs to be further investigated in future clinical trials.

## 6 CONCLUSIONS

NPC is a complex disease whose development and progression are influenced by genetic factors, immune activities, environmental dynamics, and EBV infection. Over the past few decades, there has been a gradual decline in the incidence and mortality rates of NPC largely due to a more detailed understanding of the pathogenesis of NPC and the development of precision medicine, including advancements in the screening and treatment of this disease. As we now have a more thorough understanding of how EBV interacts with epithelial cells, what signaling pathways mediate NPC aggressiveness and how these cancer cells escape immune surveillance and modify the tumor microenvironment, we will be able to develop more effective vaccines for cancer prevention, better screening tools, and more effective treatment with less toxicity.

## CONFLICTS OF INTERESTS

The authors declare no competing interests.
